# Design and immunological evaluation of two-component protein nanoparticle vaccines for East Coast fever

**DOI:** 10.3389/fimmu.2022.1015840

**Published:** 2023-01-13

**Authors:** Anna Lacasta, Hyung Chan Kim, Elizabeth Kepl, Rachael Gachogo, Naomi Chege, Rose Ojuok, Charity Muriuki, Stephen Mwalimu, Gilad Touboul, Ariel Stiber, Elizabeth Jane Poole, Nicholas Ndiwa, Brooke Fiala, Neil P. King, Vishvanath Nene

**Affiliations:** ^1^ Animal and Human Health program, International Livestock Research Institute (ILRI), Nairobi, Kenya; ^2^ Department of Biochemistry, University of Washington, Seattle, WA, United States; ^3^ Institute for Protein Design, University of Washington, Seattle, WA, United States; ^4^ Summer Undergraduate Research Fellowship Program, Caltech, Pasadena, CA, United States; ^5^ Research Methods Group, International Livestock Research Institute (ILRI), Nairobi, Kenya

**Keywords:** protein nanoparticles, nanoparticle vaccines, livestock vaccines, tick-borne disease, East Coast fever, cattle, *Theileria parva*, *Rhipicephalus appendiculatus*

## Abstract

Nanoparticle vaccines usually prime stronger immune responses than soluble antigens. Within this class of subunit vaccines, the recent development of computationally designed self-assembling two-component protein nanoparticle scaffolds provides a powerful and versatile platform for displaying multiple copies of one or more antigens. Here we report the generation of three different nanoparticle immunogens displaying 60 copies of p67C, an 80 amino acid polypeptide from a candidate vaccine antigen of *Theileria parva*, and their immunogenicity in cattle. p67C is a truncation of p67, the major surface protein of the sporozoite stage of *T. parva*, an apicomplexan parasite that causes an often-fatal bovine disease called East Coast fever (ECF) in sub-Saharan Africa. Compared to I32-19 and I32-28, we found that I53-50 nanoparticle scaffolds displaying p67C had the best biophysical characteristics. p67C-I53-50 also outperformed the other two nanoparticles in stimulating p67C-specific IgG1 and IgG2 antibodies and CD4^+^ T-cell responses, as well as sporozoite neutralizing capacity. In experimental cattle vaccine trials, p67C-I53-50 induced significant immunity to ECF, suggesting that the I53-50 scaffold is a promising candidate for developing novel nanoparticle vaccines. To our knowledge this is the first application of computationally designed nanoparticles to the development of livestock vaccines.

## 1 Introduction

Recent technological innovations in protective epitope identification, structure-based antigen design, mRNA, viral-vectored and nanoparticle-based vaccine delivery platforms are enabling new approaches to vaccine design (1). These advances, coupled with a deeper understanding of vaccine-elicited immunity obtained from, for example, systems approaches (2, 3), have propelled vaccine-related research into a new and unprecedented era. However, as a field, livestock vaccinology tends to slowly adopt novel technologies. Market size considerations and the existence of vaccines against the major high-intensity livestock production diseases in high-income countries significantly influence whether global animal health companies undertake product development, as the cost of vaccines is usually borne by farmers. Hence, several livestock diseases, especially in low- and middle-income countries (LMIC), remain neglected problems and many existing vaccines, such as those based on older technologies like inactivated or tissue-culture passaged live attenuated pathogens, remain as suboptimal disease control tools (4).

Among the novel technologies that are currently revolutionizing vaccinology, the structure-based design of self-assembling nanoparticle immunogens has become a key tool for enhancing vaccine-elicited immunity. Several naturally occurring nanoparticles, such as ferritin, encapsulin, and lumazine synthase (5), have been used to increase the potency of antibody responses against complex antigens such as oligomeric viral glycoproteins. Simultaneously, the emergence of computational methods for designing new self-assembling protein nanoparticles with atomic-level accuracy has enabled the design of nanoparticle scaffolds with structural features tailored to specific applications (6–11). Over the last few years, computationally designed nanoparticles have been used to display several antigens *via* both genetic fusion and molecular adaptors such as SpyCatcher/SpyTag (12, 13). Nanoparticle immunogens produced by both methods have elicited significantly more potent antibody responses than soluble antigen (14–25). Furthermore, the two-component nature of many computationally designed nanoparticles—that is, their construction from two distinct protein subunits—enables their assembly *in vitro* from independently purified proteins through simple stoichiometric mixing (7, 9, 26). This control over the assembly process allows, among other things, the controlled co-display of multiple antigenic variants, an approach that has been used to design nanoparticle vaccines that provide broadly protective immunity against influenza viruses and coronaviruses (19, 23).

*Theileria parva* is an intracellular apicomplexan parasite closely related to *Plasmodium* and *Babesia*, and is the causative agent of East Coast fever (ECF), a leading tick-borne disease of cattle in sub-Saharan Africa (27). ECF causes a notable economic burden of approximately $596 million USD annually and over one million cattle die each year as a result of *T. parva* infection (28). The disease can manifest as mild, moderate and severe (usually fatal) clinical disease and can have devastating impacts on the productivity of smallholder cattle farming by reducing meat and milk yields (29). The only commercial vaccines for control of ECF are based on an infection and treatment method (ITM) of immunization developed in the early 1970s. ITM depends on infection of cattle with a potentially lethal dose of cryopreserved sporozoites and simultaneous treatment with long-acting oxytetracycline (reviewed in (30)). Although effective, ITM vaccines are expensive, non-trivial to produce, require liquid nitrogen storage and antibiotic treatment, and require specialized personnel for its administration. Moreover, the immunity achieved with ITM is strain-specific. Thus, the presence of a wildlife reservoir of *T. parva* in buffalo, which is often immunologically distinct from cattle-derived parasite, further complicates control of *T. parva*. Novel ECF vaccines, especially those that simplify vaccine production and delivery, are highly desirable. Several candidate *T. parva* subunit vaccine antigens have been identified, including those that are the targets of schizont-infected lymphocyte-specific CD8^+^ T-cells generated by the ITM vaccine and others that are the targets of sporozoite neutralizing antibodies (reviewed in (31)).

Recent subunit vaccine development efforts targeting sporozoite antigens have focused on p67C, an 80 amino acid section from the C-terminal end of p67, a major cell surface molecule of sporozoites, as p67C is more stable and easier to express than full-length p67, and can also induce immunity to ECF (32–34). However, both p67 and p67C are poorly immunogenic and need 3 doses of 450 µg of soluble antigen to generate a protective immune response. As a result, several efforts have begun to assess whether the immunogenicity of p67C can be improved using nanoparticle technologies. For example, we have evaluated bovine immune responses to soluble p67C (s-p67C) and p67C displayed on virus-like particles (VLPs) *via* genetic fusion to hepatitis B core antigen (HBcAg-p67C) or adsorbed to hollow silica-based nanoparticles (SV-p67C). Both s-p67C and SV-p67C primed similar levels of p67C-specific antibodies and CD4^+^ T-cell proliferative responses, while SV-p67C primed a stronger IFN-γ CD4^+^ T-cell response than s-p67C. HBcAg-p67C primed a 2-3 fold higher p67C-specific antibody response, but with very little p67C-specific T-cell responses (35). The antibody data support the notion that display of antigen in ordered arrays on VLPs elicits a more robust antibody response than non-structured particulate antigen, probably due to enhanced B-cell activation by the former (36). In challenge experiments, immunization of cattle with a combination of the two nanoparticle immunogen formats, HBcAg-p67C and SV-p67C, resulted in an antigen dose-sparing effect while providing the same level of protection to ECF as s-p67C, despite a stronger challenge (35).

To enhance the design of vaccines for controlling ECF, here we studied the capacity of three computationally designed two-component nanoparticle scaffolds to display p67C. We found that a nanoparticle immunogen based on the I53-50 scaffold outperforms the other two nanoparticles, as well as HBcAg-p67C VLPs, in stimulating p67C-specific immune responses in cattle, and provides superior protection against ECF compared to s-p67C.

## 2 Materials and methods

### 2.1 Expression and purification of assembled nanoparticles

For co-expression in *E. coli*, synthetic genes encoding both nanoparticle components in a bicistronic operon were cloned into the pET29b+ vector (Novagen) using the *NdeI* and *XhoI* restriction sites (**Table S1**). Protein was expressed *via* IPTG induction in Lemo21 or BL21*(DE3) cells, using 1 L of Luria Bertani (LB) broth at 37°C for 3 hours. Cell cultures were centrifuged at 4,000 *g* for 20 minutes. Cell pellets were stored at -20°C. Cell pellets were thawed at room temperature and subsequently resuspended in 25 mL of lysis buffer (50 mM Tris pH 8, 500 mM NaCl, 0.75% CHAPS, 1 mM DTT, 50 μg/mL DNase, 50 μg/mL RNase) and homogenized prior to microfluidization. Lysates were centrifuged at 20,000 *g* for 20 minutes at 4°C. Clarified lysates were then applied to 5 mL Ni^2+^-NTA gravity columns. The resin beds were washed with 5 column volumes of wash buffer (50 mM Tris pH 8, 500 mM NaCl, 0.75% CHAPS, 1 mM DTT, 30 mM imidazole) before eluting the nanoparticle components with 4 column volumes of elution buffer (50 mM Tris pH 8, 500 mM NaCl, 0.75% CHAPS, 1 mM DTT, 500 mM imidazole). Finally, IMAC elutions were concentrated to ~1-2 mL and injected onto a Superose 6 10/300 GL AKTA FPLC column (Cytiva) to remove any remaining host cell protein, using 50 mM Tris pH 8, 150 mM NaCl as buffer. The assembled nanoparticles eluted from the column around 10-12 mL. Pure nanoparticle peak fractions were pooled for analysis and stored at -80°C.

### 2.2 Expression and purification of individual nanoparticle components

All p67C-nanoparticle trimer gene fusions were cloned into the pET29b+ vector using the *NdeI* and *XhoI* restriction sites (**Table S1**). These proteins, along with each complementary nanoparticle component (pentameric I53-50B.4PT1, dimeric I32-19B, and dimeric I32-28B), were separately expressed by IPTG induction in Lemo21 or BL21*(DE3) cells in 1 L of LB at 37°C for 3 hours, with the exception of I53-50B.4PT1, which was expressed at 18°C for 5 hours. Cell pellets were harvested by centrifugation at 4°C for 20 min at 4,000 *g* and stored at -20°C. Cell pellets were thawed at room temperature and lysed and IMAC-purified in the same manner as in the co-expressed nanoparticle production section above, using either gravity columns or a 40 mL Ni^2+^-NTA column on an AKTA FPLC (Cytiva). Lastly, IMAC elution fractions were concentrated using 10 kDa MWCO spin filters (Amicon, Sartorius) to ~10 mL for injection onto a HiLoad 16/60 Superdex 200 pg size exclusion chromatography column on an AKTA FPLC (Cytiva) as a polishing step to remove any higher order oligomers or remaining host cell protein. The SEC buffer contained 50 mM Tris pH 8, 500 mM NaCl, 0.75% CHAPS.

### 2.3 *In vitro* assembly of nanoparticles

Purified nanoparticle components were mixed at an equimolar ratio, usually ranging from ~25-50 μM, adding additional buffer to *q.s.* to the desired volume (usually 1-2 mL). Samples were incubated at room temperature for at least 30 min with gentle rocking prior to removing any insoluble aggregate that may have formed with a 0.2 μm filter followed by SEC on a Superose 6 10/300 GL column (Cytiva) (p67C-I32-19 and p67C-I32-28) or Superose 6 prep grade XK 50/60, 55 cm column (Cytiva) (p67C-I53-50) to remove any soluble higher order oligomers or unassembled residual component. The SEC running buffer consisted of 50 mM Tris pH 8, 250 mM NaCl, 50 mM L-Glycine.

### 2.4 Dynamic light scattering

Particle size measurements were conducted in a Nano-DSF (Unchained Laboratories) and data collected at 25°C. Briefly, sample was applied to quartz capillaries in a cassette and ten acquisitions of 5 seconds were obtained, using auto-attenuation of the laser.

### 2.5 Negative stain electron microscopy

p67C nanoparticles were diluted to 75 μg/mL in 50 mM Tris pH 8, 250 mM NaCl, and 50 mM glycine prior to application of 3 μL of sample onto freshly glow-discharged 300 mesh copper grids. Next, nanoparticles were stained onto grids one of two ways (1): p67C-I53-50 sample was incubated for 1 minute before the grid was dipped in a 50 μL droplet of water and excess liquid blotted away with filter paper. The grids were then dipped into 6 μL of 0.75% w/v uranyl formate stain. Stain was blotted off with filter paper (Whatman), then the grids were dipped into another 6 μL of stain and incubated for ~70 s (2). p67C-I32-19 and p67C-I32-28 samples were incubated for ~30 s before the grids were blotted by filter paper and immediately 3 μL of 0.75% w/v uranyl formate stain was applied onto each grid. Incubation and stain application was repeated two more times. Finally, the stain was blotted away, and the grids were allowed to dry for at least 5 min. The samples were imaged in a Talos model L120C electron microscope at 45,000×.

### 2.6 Soluble p67C expression

Bulk production and purification of s-p67C was outsourced to GenScript Biotech Corporation as previously described (35). Briefly, residues 572-651 of *T. parva* (Muguga) p67 antigen were cloned into pET-28a+ (Novagen) and expressed as a 114-residue fusion protein, of which the terminal 80 residues encode p67C, affinity purified under denaturing conditions and extensively dialyzed against PBS followed by 0.22 μm filter sterilization and storage at -80°C. As judged by SDS-PAGE, the protein was greater than 95% pure.

### 2.7 Cattle immunogenicity and experimental vaccine trial experiments

Holstein/Friesian and Ayrshire cattle (*Bos taurus*) from 6 to 9 months old and negative for *T. parva* antibodies as determined by ELISA (37) were sourced from farms in the Kenyan highlands from areas free of ECF. Animals were vaccinated against Foot-and-Mouth Disease (FMD) using a quadrivalent vaccine three weeks before the start of the experiment. All animals were clinically assessed by the ILRI Institutional Veterinarian before the start of the experiment and only healthy animals were enrolled in the experiment. Animal experiments and routine maintenance was in accordance with procedures approved by ILRI’s Institute Animal Care and Use Committee (IACUC experiment references 2016.15, 2017.08 for immunogenicity studies and 2019.02 for the challenge experiment).

For the nanoparticle immunogenicity studies, three animals were randomly assigned to one of five experimental groups and each animal received three doses of antigen, with 28-day intervals between booster doses (**Table 1**). Animals received a total 2 mL per inoculation, administered subcutaneously in the neck, and were monitored for adverse clinical reactions at the site of inoculation. All immunogens were diluted in PBS and mixed with Montanide ISA 206 VG adjuvant (Seppic) in a 1:1 ratio following the manufacturer’s instructions. Group 1 animals (BN035, BN050 and BN064) were immunized with 451 μg/dose of p67C-I32-19; Group 2 (BN049, BN055 and BN068) were immunized with 366 μg/dose p67C-I32-28, Group 3 (BN043, BN054 and BN058) were immunized with 424 μg/dose of p67C-I53-50; Group 4 animals (BM005, BM062 and BM065) received 100 μg/dose of soluble p67C; and Group 5 animals (BM135, BM162 and BM203) were immunized with 300 μg/dose of HBcAg-p67C (Group 4 and 5 animals groups were already described in (35). All animals received an equivalent of 70 μg/dose of p67C. None of the animals exhibited immediate or delayed hypersensitivity, indicating safety of the immunogen formulations. Samples of blood for preparation of serum and peripheral blood mononuclear cells (PBMCs) were taken at various times as indicated in the text.

**Table 1:** Summary of immunogenicity and challenge experiment animal groups and immunogens.

Group	Immunogen	Doses	Adjuvant	p67C/inoculation (µg/dose)	Total protein (µg/dose)	Number of animals	Challenge
**Group 1**	p67C-I32-19	3	ISA 206 VG	70	451	3	N/A
**Group 2**	p67C-I32-28	3	ISA 206 VG	70	366	3	N/A
**Group 3**	p67C-I53-50	3	ISA 206 VG	70	424	3	N/A
Group 4*	s-p67C	3	ISA 206 VG	70	100	3	N/A
Group 5*	HBcAg-p67C	3	ISA 206 VG	70	300	3	N/A
**Group 6**	p67C-I53-50	3	ISA 206 VG	140	847	15	LD70
**Group 7**	N/A	N/A	N/A	N/A	N/A	15	LD70

*Previously reported in Lacasta et al., 2021 (35).

For the experimental vaccine trial, thirty cattle were allocated in two groups of 15 each (**Table 1**). Group 6 animals received three doses (with 28-day intervals between booster doses) of 847 μg/dose of p67C-I53-50 (equivalent to ~140 μg of p67C), formulated with ISA 206 VG. Group 7 animals were kept unvaccinated to be used as a control group to estimate the sporozoite challenge dose in the cohort of cattle. Twenty-one days after the last boost, all animals were given a subcutaneous syringe challenge of 1 mL of *T. parva* Muguga sporozoites (stabilate #3087), as previously described (35). After the challenge, all experimental cattle were monitored daily for changes in rectal temperatures and other clinical manifestations of ECF, which were used to calculate an ECF score from 0 to 10 (38). The score was used to define whether animals were susceptible to ECF, a score of 6 and above, or immune to ECF, a score of 5.99 and below. Serum samples and peripheral blood mononuclear cells (PBMCs) were taken for analyses at various times as indicated in the text.

### 2.8 Quantitation of IgG anti-p67C antibodies

The quantitation of p67C-specific antibodies (in μg/mL) present in serum samples was measured by extrapolating from a standard curve, built with affinity purified p67C-specific antibodies as described in (39). A reference pool of anti-p67C antibodies was generated by mixing 2 mL serum from each animal in the immunogenicity studies: BM135, BM162 and BM203 immunized with HBcAg-p67, BN046, BN047 and BN048 immunized with HBcAg-p67C + SV-p67C (35), BM005, BM062 and BM065 immunized with s-p67C (35) and BN043, BN054 and BN058 immunized with p67C-I53-50 (this study, group 3), at day 77. Briefly, total IgG was isolated from the pooled sera using protein G resin (Pierce) following the manufacturer’s instructions. p67C-specific antibodies were then purified from the total IgG using NHS-activated Sepharose 4 Fast Flow (Cytiva) coupled with s-p67C in a 2:1 ratio, following the manufacturer’s instructions. About 5% of 15 mg IgG was recovered as p67C-specific antibodies (0.76 mg). Antibody concentrations were determined using the Qubit Protein Assay and used to generate a standard curve for the ELISA with antibody concentrations ranging from 250 to 1.95 ng/mL on plates coated with s-p67C.

Maxisorp 96-well plates (Nunc) were coated with 0.5 μg/mL of s-p67C diluted in PBS and incubated at 4°C overnight. After blocking the plates for an hour at 37°C with 1% bovine serum albumin (BSA, Merk) and 0.1% Tween20 (Merk) diluted in PBS (blocking buffer), sera diluted at 1/10,000 and 1/100,000 in blocking buffer were added to the plate and incubated 2 h at 37°C. The presence of p67C-specific antibodies was detected with mouse anti-bovine IgG : HRP clone IL-A2 (Bio-Rad) diluted at 1 μg/mL in the blocking buffer and incubated for 1 h at 37°C. The reaction was developed with 50 μl of TMB plus 2 (Kem-En-Tec Diagnostics) in the dark for 10 minutes at room temperature and stopped using 50 μL of 0.5 M H_2_SO_4_ (Honeywell-Fluka). Plates were washed four times in between each step using PBS containing 0.05% Tween20 and assays were carried out in duplicates. The optical density at 450 nm was read using Synergy HT ELISA reader (BioTek Instruments) and four-parameter fits were generated from standard curve values with GraphPad Prism v8 (GraphPad software). This curve was used to convert absorbance of diluted samples into concentration of p67C-specific antibodies. The results are expressed as μg/mL of p67C-specific antibodies after correcting with the dilution factor.

### 2.9 Measurement of p67C-specific IgG1 and IgG2, IgG specificity to overlapping p67C synthetic peptides and sporozoite neutralization assay

p67C-specific IgG1 and IgG2 subtype responses were assessed by means of ELISA, as previously described (33) Briefly, plates were coated with 0.5 μg/mL of p67C diluted in PBS. Serum samples were 2-fold diluted in blocking buffer from 1/100 to 1/12,800 and developed with sheep anti-bovine IgG1 and IgG2 HRP-conjugated (Bio-Rad). Half-max antibody titers were calculated using OD values from an antibody dilution series with GraphPad Prism v8 (GraphPad software). To further characterize the antibody response, sporozoite neutralization assays and the specificity to 10 overlapping 15-mer peptides to p67C were also measured as previously described with slight differences (33, 35). The 15-mer peptide sequences (**Supplementary Table S2**) correspond to peptide 73 to 82 in a Pepscan screen of antibody responses to full length p67 (40), with the exception that the peptides were biotinylated and captured on Immobilizer Streptavidin clear plates (Nunc). Serum samples were diluted at 0.1 μg/mL of p67C-specific antibodies using blocking buffer. Negative bovine serum was added to the diluted samples to adjust the volume of bovine sera present in the reaction. The results presented are the percentage of the signal for a particular peptide compared to the sum of ODs for all peptides for a particular sample (% signal sample X peptide X = OD sample X peptide X/sum ODs sample X all peptides × 100).

### 2.10 CD4^+^ T-cell ^3^H-thymidine proliferation assay and IFN-γ ELISpot

Purified CD4^+^ T-cell responses were analyzed by IFN-γ ELISpot and ^3^H-thymidine CD4^+^ T-cell proliferation assays as previously described (33, 35). The stimuli used in both assays were: s-p67C at 20 μg/ml (equivalent to 2.5 μM) or a pool of 25-mer synthetic peptides at 2 μM that overlap by 16 amino acid residues covering the p67C sequence (Mimotopes Pty.). Media was used as a general negative control, and ovalbumin (Merk) and 25-mer peptides not related to *T. parva* (Mimotopes Pty.) were used as negative controls. ConA at 2.5 μg/mL was used as a positive control.

### 2.11 Statistical analysis

Differences among Groups 1, 2, 3, 4 and 5 immune parameters were assessed using a linear regression with a group fixed effect, and multiple comparisons made with Holm adjustment. Immune parameters assessed were natural log transformed antibody titers at day 77, percentage of sporozoite neutralization and CD4^+^ T-cell responses (in R, package: stats). Residual checks were carried out on all models to confirm model assumptions.

Differences in ECF scores between the two challenged groups (group 6 and 7) was assessed using a permutation test of independence (in R, package: coin), that provides an asymptotic exact distribution using the quasi-MonteCarlo method. Protection for the two groups was compared using a Fisher’s exact test (in R, package: stats) (41).

## 3 Results

### 3.1 Production and characterization of p67C nanoparticle immunogens

**Figure 1:**
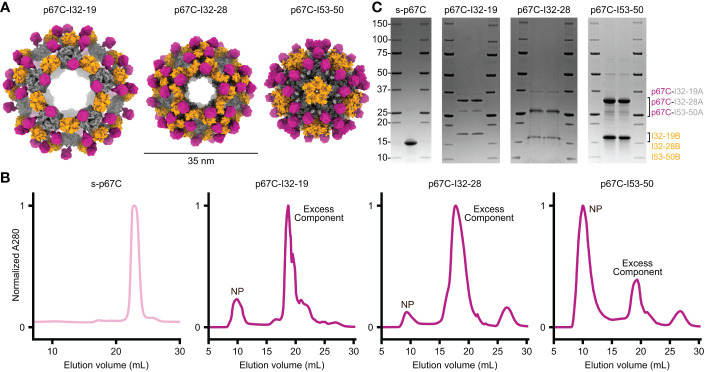
Design and purification of two-component nanoparticles displaying p67C co-expressed *in vivo*. **(A)** Design models of three distinct icosahedral nanoparticles displaying p67C, shown to scale. **(B)** Preparative size exclusion chromatography of s-p67C antigen and nanoparticles displaying p67C. Clear peaks were observed at the expected retention times for soluble antigen and nanoparticles (NP); excess component peaks were also observed for the nanoparticles. **(C)** SDS-PAGE of soluble p67C antigen and nanoparticles displaying p67C after purification by SEC. Bands were observed at the expected molecular weights for the two components of each nanoparticle. The left and right lanes for each nanoparticle are samples pre- and post-freeze/thaw, respectively.

Recent studies have demonstrated that weakly immunogenic monomeric proteins can be improved by multivalent display on protein nanoparticles (23). We displayed the p67C antigen on three computationally designed, two-component protein nanoparticle scaffolds with icosahedral symmetry: I53-50, I32-28, and I32-19 (9). I53-50 is constructed from 20 trimeric (I53-50A) and 12 pentameric (I53-50B) building blocks, while I32-28 and I32-19 each contain 20 trimeric (I32-28A, I32-19A) and 30 dimeric (I32-28B, I32-19B) components such that each nanoparticle comprises a total of 120 subunits. We genetically fused p67C to the N-terminus of each trimeric component so that 60 copies of the antigen are displayed in a repetitive array on each nanoparticle exterior (**Figure 1A**). The amino acid sequences of all novel proteins used in this study are provided in **Supplementary Table S1**.

We co-expressed each pair of nanoparticle components in *E. coli* in a bicistronic format (9) and extracted pre-assembled nanoparticles from *E. coli* lysates using immobilized metal affinity chromatography (IMAC) and size-exclusion chromatography (SEC). In parallel, we expressed and purified soluble p67C as a monomeric antigen (s-p67C). The SEC chromatogram of s-p67C yielded a single major peak at the expected elution volume, while the chromatograms of the nanoparticle immunogens all contained peaks at the expected elution volume as well as peaks corresponding to residual, unassembled components (**Figure 1B**). The ratio of the two peaks varied across the three nanoparticles, with p67C-I53-50 yielding the highest ratio of nanoparticle to component peaks and p67C-I32-28 the lowest, suggesting that p67C-I53-50 assembled to form the intended icosahedral nanoparticle more efficiently than the other two immunogens. SDS-PAGE of s-p67C and all three nanoparticles revealed bands at the expected molecular weights, with no degradation observed after a single freeze-thaw cycle (**Figure 1C**). Expression of p67C epitopes on the nanoparticles was confirmed using bovine anti-p67C polyclonal sera and a murine sporozoite-neutralizing mAb, AR21.4, in immunoblots and ELISA, respectively (data not shown). However, we were unable to obtain information on the conformation of p67C on the nanoparticles as AR21.4 binds to a linear epitope (40) and no conformation-specific mAbs for p67C have been reported.

### 3.2 Comparison of anti-p67C antibody responses induced by p67C-nanoparticles in *Bos taurus* cattle

**Figure 2:**
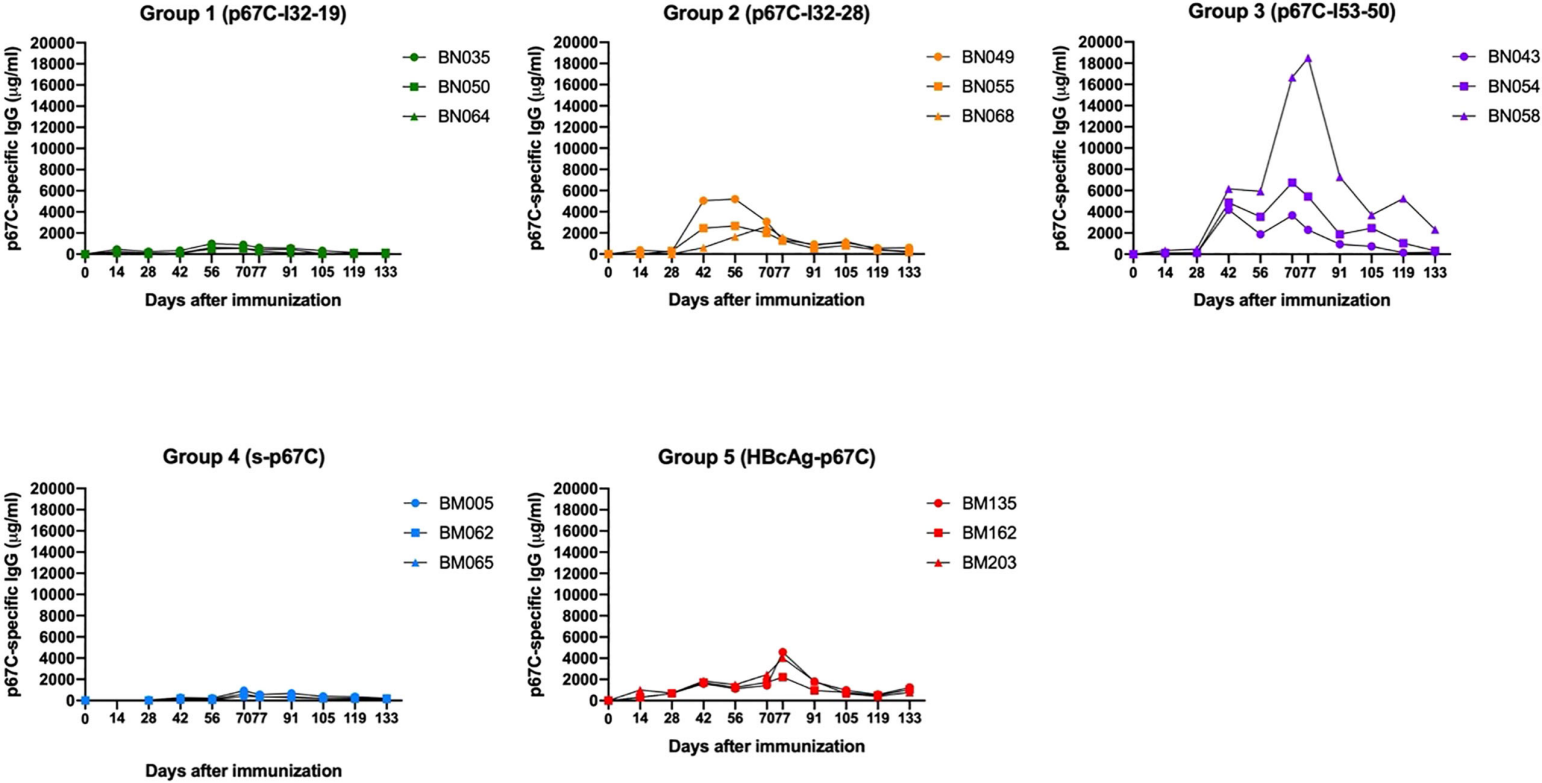
Kinetics of anti-p67C specific total IgG levels from day 0 to day 133, approximately two and a half months after the third antigen dose. Antibody titers from cattle immunized with p67C-I32-19 (Group 1), p67C-I32-28 (Group 2), p67C-I53-50 (Group 3), s-p67C (Group 4) or HBcAg-p67C (Group 5); from previous experiments (35), are presented.

To assess the immunogenicity of the different p67C nanoparticles, three groups of three cattle were immunized with a three-dose regimen of p67C nanoparticle formulated with ISA 206 VG as adjuvant and measured as p67C-specific IgG in μg/mL. The immune responses in this cohort of cattle (**Figure 2** and **Table 2**) were compared with the responses of two other groups of three cattle immunized with s-p67C or HBcAg-p67C given the same dosing regimen, which were described in an earlier publication (35). The half-max p67C-specific ELISA data from the latter was re-quantified as p67C-specific IgG in μg/mL. This newly developed ELISA permits a more accurate comparative analysis of anti-p67C antibody responses between time points, individual animals, and experiments (**Supplementary Table S3**). It is apparent that cattle immunized with p67C-I53-50 nanoparticles gave the highest antibody response (**Figure 2**).

**Table 2:** Summary of antibody titers for immunogenicity studies in cattle.

Group	Animal ID	p67C-specific IgG (µg/ml)		Ratio IgG1/IgG2 (day 77)	Half-max p67C-specific IgG1 (day 77)	Half-max p67C-specific IgG2 (day 77)
		Day 77	Day 133			
Group 1 (p67C-I32-19)	BN035	607.02	60.38	2229	2229	0
	BN050	412.43	148.45	1569	1569	0
	BN064	251.59	27.95	1384	1384	0
Group 2 (p67C-I32-28)	BN049	1342.16	593.07	4	6316	1469
	BN055	1255.10	198.43	14	3122	229
	BN068	1596.97	236.57	10	9029	938
Group 3 (p67C-I53-50)	BN043	2292.79	191.96	68	6528	96
	BN054	5431.88	317.51	9	11928	1333
	BN058	18508.03	2319.06	10	27967	2934
Group 4 (s-p67C)	BM005	556.31	210.42	3,207*	3,207*	Not detected*
	BM062	347.94	184.29	2,600*	2,600*	Not detected*
	BM065	343.14	145.52	12*	3150*	255*
Group 5 (HBcAg-p67C)	BM135	4569.33	1240.93	7*	12274*	1,682*
	BM162	2209.45	1041.57	15*	11,345*	753*
	BM203	4018.22	790.38	8*	16,394*	2,018*

*Previously reported in Lacasta et al., 2021 (35).

Antibody levels at day 77 are from a time point of particular interest, as this is when cattle would be given a sporozoite challenge in experimental vaccine trials (33–35). At this timepoint, p67C-I50-53 induced the highest anti-p67C antibody response, followed by p67C-I32-28 (p < 0.05), while the performance of p67C-I32-19 was equivalent to s-p67C (**Table 2**). The anti-p67C antibody responses varied from a low of 252 μg/mL to a high of 18,508 μg/mL. HBcAg-p67C was the second-best immunogen and performed better than p67C-I32-28, p67C-I32-19 and s-p67C. Boosting of antibody responses was most evident in cattle immunized with p67C-I53-50 (**Figure 2**). At day 133, most anti-p67C antibody levels were substantially reduced compared to the peak at day 77 (**Supplementary Table S3**).

**Figure 3:**
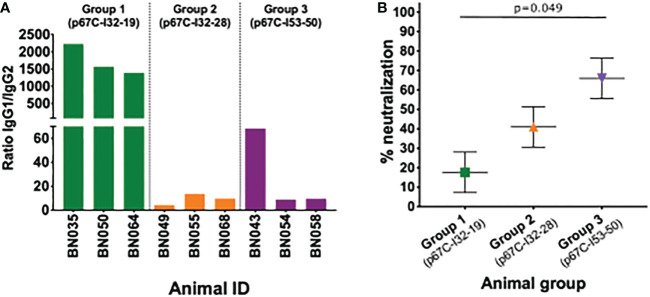
IgG subtypes and neutralizing capacity of sera. **(A)** Detection of IgG subtypes at day 77 is expressed as the ratio of IgG1 to IgG2 anti-p67C responses. **(B)** Neutralization capacity of serum of animals immunized with p67C-I32-19 (Group 1), p67C-I32-28 (Group 2) or p67C-I53-50 (Group 3) at day 77. The results are presented as the percentage of reduction of infection compared with the pre-immunization sera from the same animals. Individual animals, mean and standard error of the mean bars are presented. Group 1 and 3 showed significant differences when applying multiple comparison with Holm adjustment after linear regression (p=0.049).

Measurement of p67C-specific half-max ELISA IgG1 and IgG2 levels at day 77 demonstrated that an antibody isotype switch to IgG2 occurred in cattle that received p67C-I53-50 and p67C-I32-28, but not in those receiving p67C-I32-19 (**Figure 3A** and **Table 2**). Similar results were observed in animals receiving HBcAg-p67C (35).

Sporozoite neutralization assays were also performed using the day 77 sera (**Figure 3B** and **Table 3**). Cattle in group 3 (p67C-I53-50) had the highest levels of neutralizing antibodies and group 1 (p67C-I32-19) the lowest (p = 0.049), mirroring the levels of total p67C-specific antibodies elicited by each immunogen. The results from group 1 and group 3 cattle were similar to those induced by s-p67C and HBcAg-p67C, respectively (35). We note that in its current format, the sporozoite neutralization assay is a qualitative rather than quantitative assay.

**Table 3:** Summary of CD4^+^ T-cell proliferation IFNg-ELISpot and seroneutralization assay results for immunogenicity studies on cattle.

Group	Animal ID	CD4^+^ proliferation index		IFNγ-SC*/million CD4^+^ (ELISPOT)		% SN**
		s-p67C	25-MER	s-p67C	25-MER	
Group 1 (p67C-I32-19)	BN035	1.28	0.68	18.49	64.73	11
	BN050	2.64	2.88	0.00	26.25	20
	BN064	9.61	2.27	7.77	0.00	22
Group 2 (p67C-I32-28)	BN049	76.84	7.44	38.16	15.26	48
	BN055	11.34	1.64	11.11	27.76	24
	BN068	31.36	14.32	24.12	9.65	51
Group 3 (p67C-I53-50)	BN043	12.61	4.05	35.19	70.38	36
	BN054	0.00	3.02	61.90	581.91	87
	BN058	145.15	86.18	96.61	135.26	75

*SC: secreting cells.

**SN: seroneutralization.

### 3.3 Measurement of bovine CD4^+^ T-cell responses to p67C induced by the p67C nanoparticles

**Figure 4:**
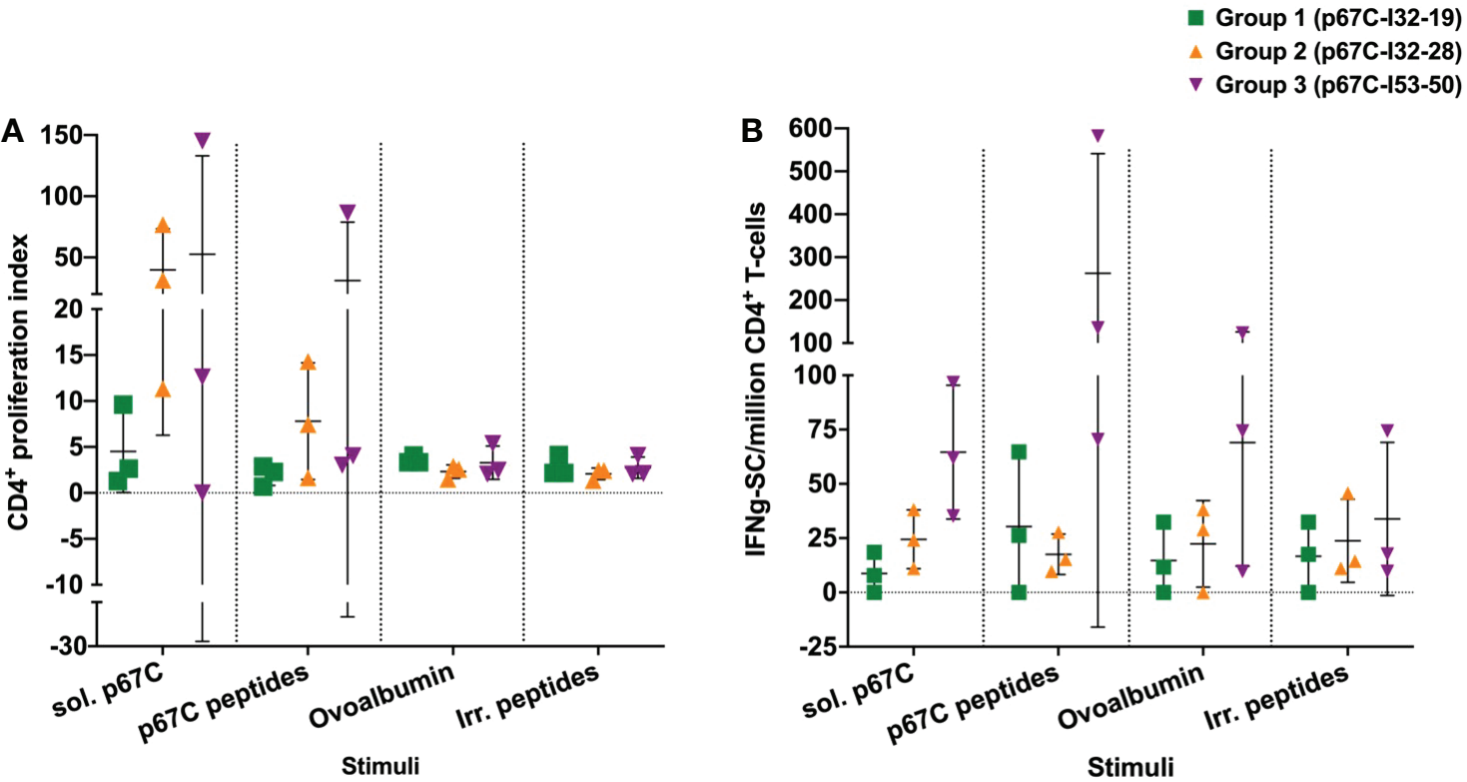
The responses of enriched CD4^+^ T-cell from immunized cattle to different stimuli were measured in cattle from immunogenicity studies. **(A)** The p67C-specific proliferative cellular response; and **(B)** the p67C-specific IFNγ-secreting cells per million of CD4^+^ T-cells in individual animals from group 1 to 3. Two different stimuli were used: soluble protein (sol. p67C) and a pool of 25-mer p67C overlapping peptides (p67C peptides). Negative stimulus responses are also included: ovalbumin and irrelevant peptides (irr. peptides). In both panels individual animals (n=3), the mean and standard error of the mean bars are shown.

An enriched population of CD4^+^ T-cells, purified from PBMCs obtained at day 70, was used to measure T-cell proliferation and IFN-γ secretion responses to s-p67C and overlapping 25-mer p67C synthetic peptides (**Figure 4** and **Table 3**). In both assays, cattle immunized with p67C-I53-50 mounted the best immune response, whereas cattle immunized with p67C-I32-19 mounted the weakest response. Cattle that received p67C-I32-28 mounted an IFN-γ response to s-p67C but not to synthetic p67C peptides. In our previous studies (35), cattle immunized with s-p67C mounted a good response in both T-cell assays, whereas the response in HBcAg-p67C immunized cattle was very weak, similar to that induced by p67C-I32-19.

### 3.4 Linear p67C peptide antibody specificity of sera from immunogenicity studies

In addition to measuring the kinetics of the global anti-p67C antibody response during the immunization regimen, we measured the ability of these sera to react with a series of ten overlapping 15-mer synthetic peptides from p67C. These peptides had been previously used in a Pepscan analysis to map sequences recognized by p67-specific murine mAbs and the reactivity of sera from cattle immunized with full length p67 (40). Serum samples from day 28, 42, 56 and 77 from each animal in this study were adjusted to a concentration of 0.1 μg/ml of anti-p67C antibodies. A re-assessment of the specificity of bovine antibodies induced by s-p67C and HBcAg-p67C described above was also included. Hence, the strength of the signal detected in the peptide ELISA heatmap should reflect the relative abundance of p67C peptide reactivity within the bovine polyclonal response.

**Figure 5:**
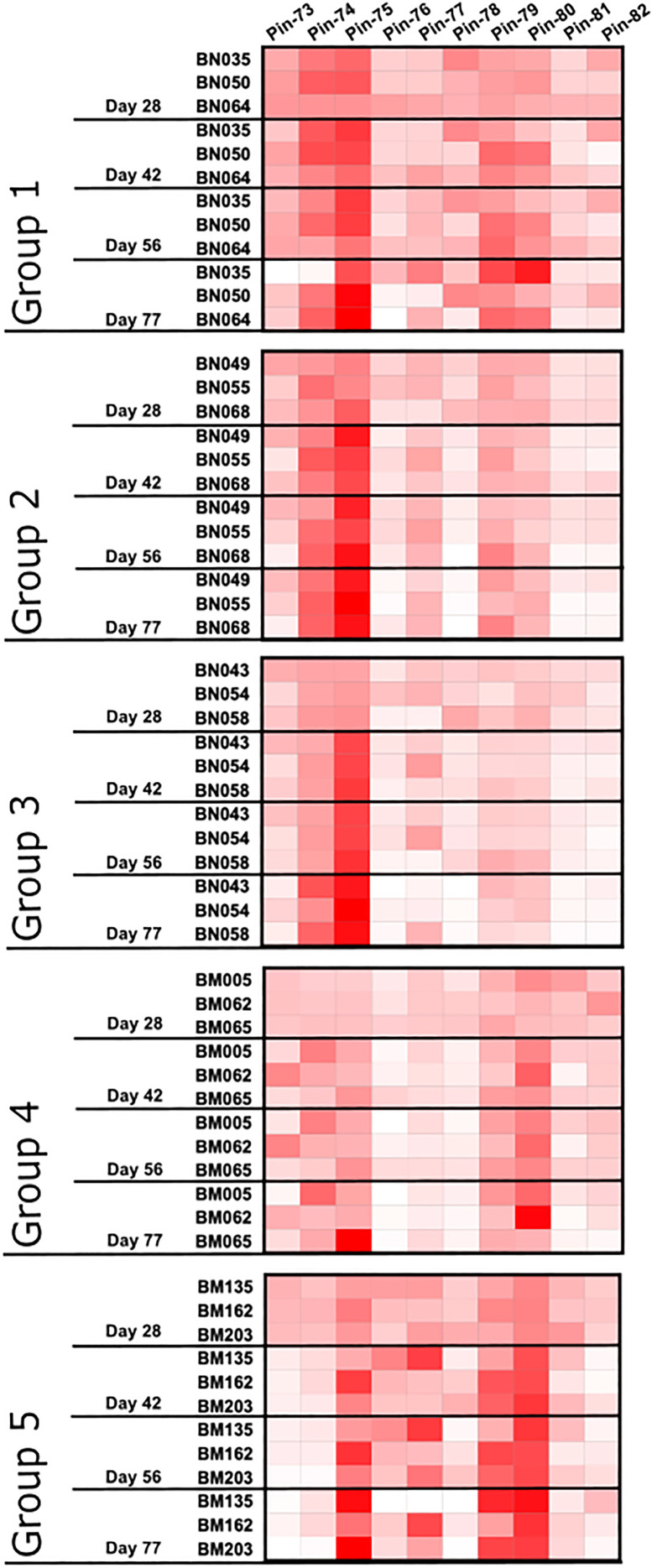
Pattern of antibody reactivity to p67C 15-mer overlapping peptides in animals subjected to immunogenicity studies. Pattern of IgG reactivity to overlapping 15-mer p67C peptides (named pins, as previously described in (40)), at day 28, 42, 56 and 77 after first dose of antigen.

For all immunogens, the immunization regimen appears to focus the antibody response following antigen boosts so that at day 77, two to four peptides constitute an immunodominant response (**Figure 5**). s-p67C sera bound to peptides 74, 75, 79 and 80. This reactivity was also seen with sera to the p67C nanoparticles, but with an increasing shift to dominance of peptide 74 and 75 reactivity with p67C-I32-28 and p67C-I53-50 sera. HBcAg-p67C sera bound to peptide 75, 77, 79 and 80.

We had previously demonstrated that sporozoite neutralizing murine mAbs AR21.4 and 1A7 bound to peptides 78 and 79 and 79 and 80, respectively, identifying the overlapping sequences of SERQPSL and PSLVITD as playing an important functional role in mAb recognition (40). Reactivity with peptide 79 and 80 was present in all sera tested, and highest in HBcAg-p67**C** and lowest in p67C-I53-50 sera. However, the antibody response to peptide 78 was very poor in all samples, except for p67C-I32-19 sera. The reactivity of most sera with peptide 75 is interesting as this peptide is recognized by the anti-p67 mAb 38.1 with no sporozoite neutralizing capacity (40).

### 3.5 Assessment of p67C-I53-50 to induce immunity to East Coast fever in *Bos taurus* cattle

Our small-scale comparative immunogenicity study indicated that the p67C-I53-50 nanoparticles induced the highest level of p67C-specific antibody, sporozoite neutralizing capacity and T-cell responses. Hence, we designed an experimental vaccine trial where a group of 15 cattle in group 6 were immunized with a dose of p67C-I53-50 containing ~140 μg of p67C antigen at weeks 0, 4 and 8. We selected this dose of antigen based on previous results where a combination of two nanoparticle immunogen formats, HBcAg-p67C and SV-p67C, each one delivering ~70 μg p67C antigen, resulted in an efficacy of 53% against an LD_90_ sporozoite challenge (35). Our intention was to test whether a single immunogen could replace a two-immunogen formulation by inducing similar levels of immunity to ECF. We observed no apparent side effects to this immunization regimen.

**Figure 6:**
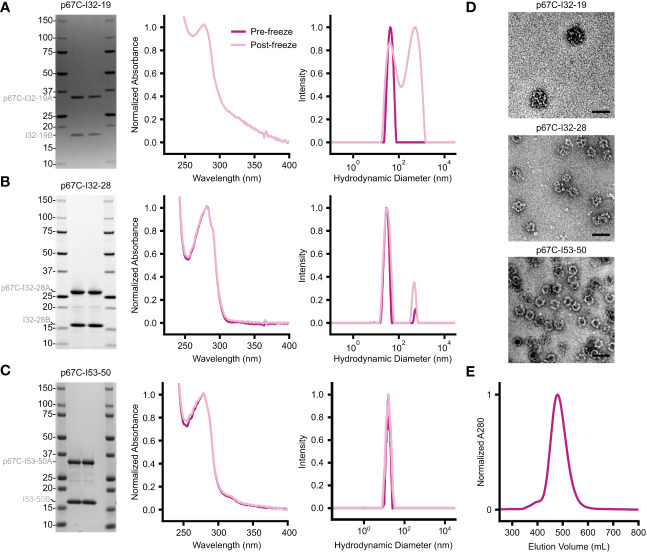
Biophysical characterization of *in vitro*-assembled two-component nanoparticles displaying p67C. **(A–C)** From left to right, SDS-PAGE, UV/vis absorbance, and DLS data are provided for **(A)** p67C-I32-19, **(B)** p67C-I32-28, and **(C)** p67C-I53-50. The left and right lanes for each nanoparticle are samples pre- and post-freeze/thaw, respectively. The DLS data for p67C-I32-28 and the DLS and UV/vis data for p67C-I32-19 suggest the presence of aggregates. **(D)** Negatively stained electron micrographs are shown for each nanoparticle immunogen after purification by SEC. **(E)** Preparative SEC of a >100 mg batch of p67C-I53-50 nanoparticle on a Superose 6 prep grade XK 50/60 column yielded a single, symmetric peak corresponding to the nanoparticle immunogen.

For this vaccine trial, we produced the p67C-I53-50 nanoparticle immunogen by *in vitro* assembly. We have found that *in vitro* assembly facilitates endotoxin removal and typically yields nanoparticles with less contaminating host cell protein. In this case, SDS-PAGE, UV/vis absorbance, DLS, SEC, and negative stain electron microscopy all indicated that *in vitro* assembly of p67C-I53-50 yielded highly pure, monodisperse nanoparticles (**Figure 6**). Although we were also successful in assembling p67C-I32-19 and p67C-I32-28 nanoparticle immunogens *in vitro*, we observed signs of aggregation following assembly, particularly for p67C-I32-19 (**Figures 6A, B**).

**Figure 7:**
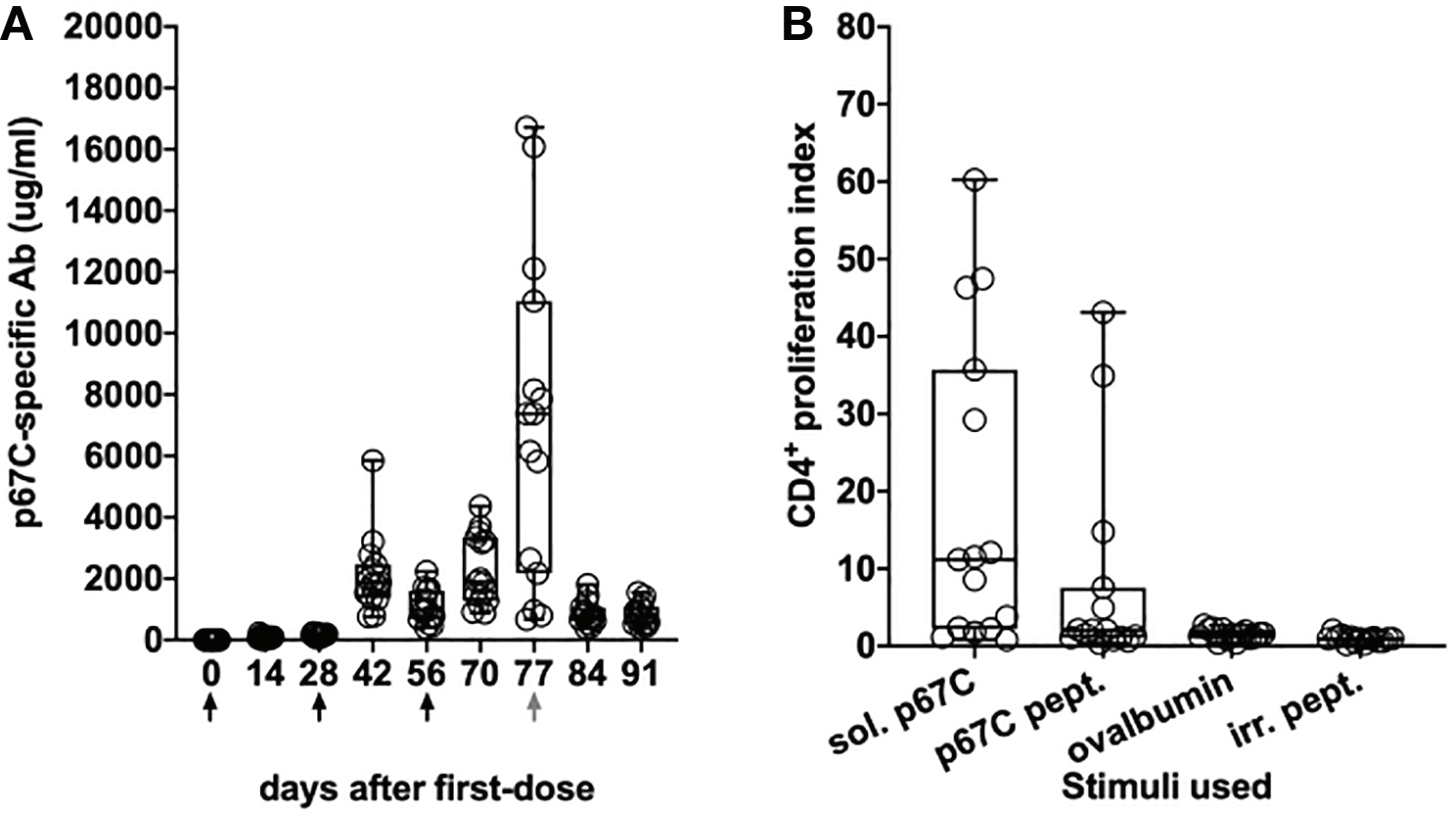
Immune responses in Group 7 (p67C-I53-50) animals. **(A)** Kinetics of Ag-specific IgG titers measured in sera. The days of antigen injection are represented by black arrows, the day of challenge is represented with a gray arrow. **(B)** p67C-specific CD4^+^ proliferation increases measured at 2 weeks after the last boost (day 70). Two different stimuli were used: soluble protein (sol. p67C) and a pool of 25-mer p67C overlapping peptides (p67C pept.). Negative stimulus responses are also included: ovalbumin and irrelevant peptides (irr. pept.). The median and the 25^th^ and 75^th^ percentile are shown as a box and the min and max values are shown with black bars. Individual animals (n=15) are also presented as black circles.

**Figure 8:**
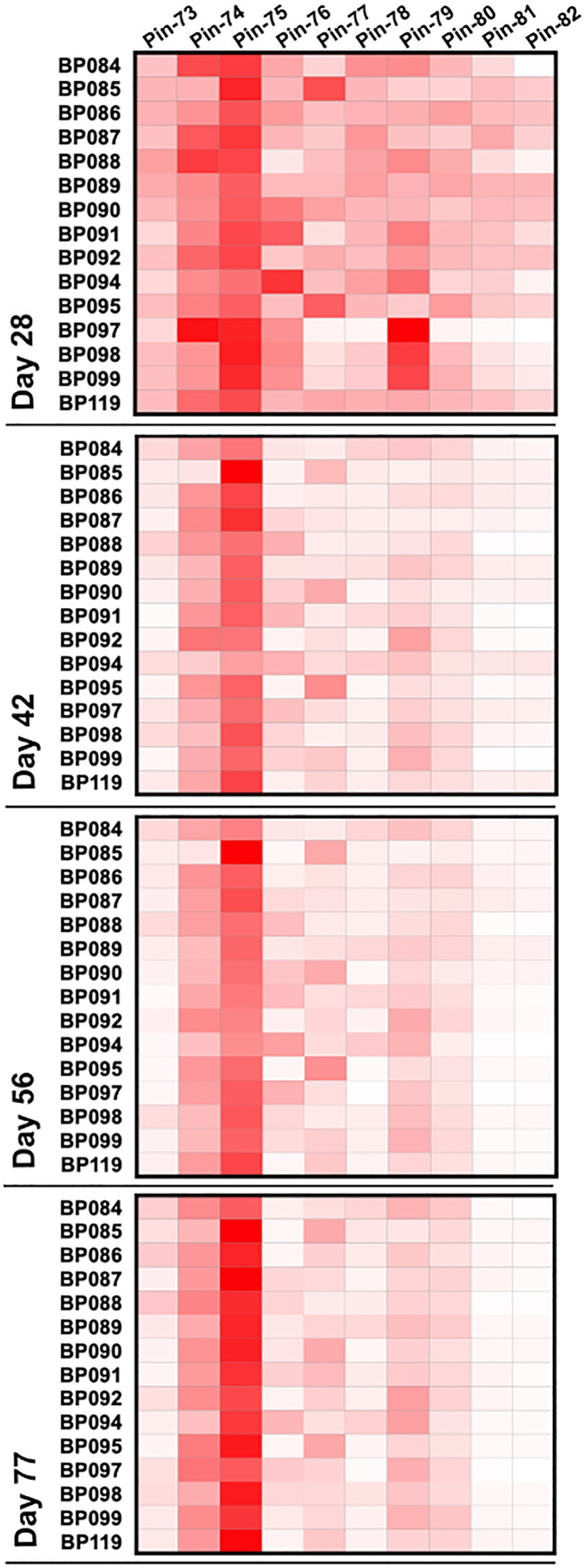
Pattern of antibody reactivity to p67C overlapping peptides in animals under challenge experiment. Pattern of IgG reactivity to overlapping 15-mer p67C peptides (named pins, as previously described in (40)), at day 28, 42, 56 and 77 after first dose of antigen.

As in the immunogenicity studies, we measured the temporal evolution of anti-p67C antibodies in μg/mL and CD4^+^ T-cell immune responses at day 70 (**Figure 7**), as well as reactivity with synthetic p67C peptides (**Figure 8**). All cattle mounted p67C-specific antibody and T-cell responses, however, there was a wider variation in this cohort of cattle than in our earlier immunogenicity study (**Table 4** and **Supplementary Table S4**). The pattern of p67C synthetic peptide reactivity was as in the immunogenicity studies and the focusing of peptide specific responses to peptide 74 and 75 after the antigen prime is more evident than in the immunogenicity study.

**Table 4:** Summary of immune parameters measured in Group 6 (challenge experiment).

Animal ID	p67C-specific IgG (µg/ml)		CD4^+^ proliferation index	
	Day 77	Day 91	s-p67C	25-MER
BP084	6150	838	60.24	43.13
BP085	16088	1231	12.08	1.07
BP086	672	570	8.56	1.14
BP087	16721	1069	29.23	1.52
BP088	12112	941	46.30	34.92
BP089	5823	508	47.46	7.54
BP090	11053	765	0.80	0.37
BP091	7380	460	11.17	2.05
BP092	7861	543	3.78	1.28
BP094	8154	450	11.53	4.91
BP095	7380	622	1.74	0.81
BP097	795	1080	35.72	14.74
BP098	957	382	2.21	0.65
BP099	2638	1539	2.34	2.10
BP119	2173	1415	1.13	2.03

Twenty-one days after the last antigen boost, all cattle in group 6 as well as 15 unvaccinated cattle (group 7) were given a needle sporozoite challenge. The latter group was included to estimate the challenge potency in this cohort of cattle, which we noticed can vary between experiments. Clinical parameters were determined as previously described (35) and the experiment was terminated on day 21 post-infection. To reduce the use of cattle, we did not include an adjuvant control as this has been previously described (34), and in this experiment an irrelevant nanoparticle control would not be expected to induce immunity to ECF.

**Figure 9:**
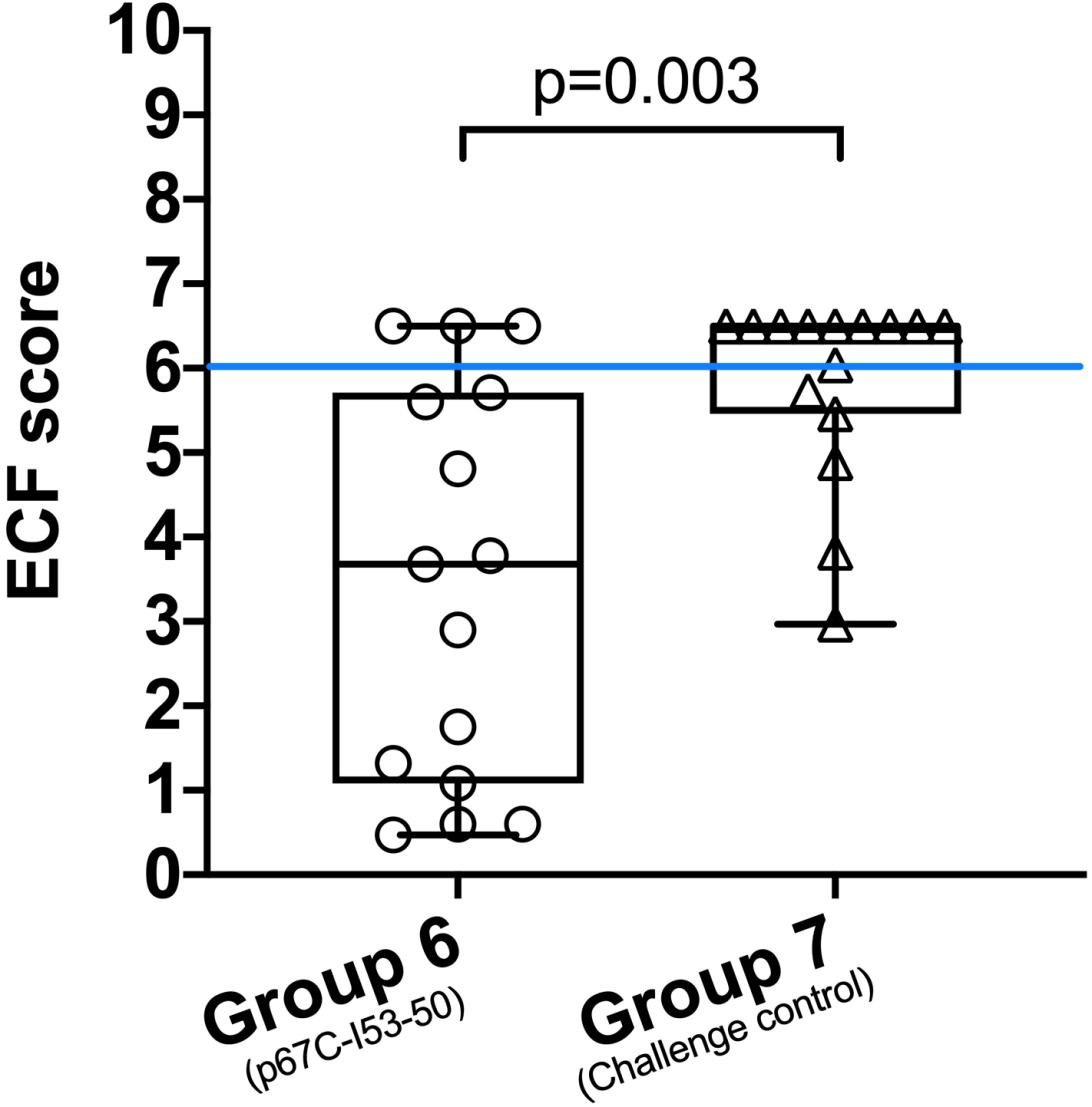
ECF scores of animals in group 6 (p67C-I53-50) and group 7 (challenge control). The median and the 25^th^ and 75^th^ percentile are shown as a box and the min and max values are shown with black bars. Individual animals are also presented (n=15). A blue line separates the protected and non-protected animals (ECF score equal or higher than 6). Using a permutation test of independence, the significance of the differences between groups is **p<0.003.

A clinical index was calculated for cattle in both groups and used to determine immune status to ECF and the sporozoite challenge dose (**Figure 9** and **Table 5**). In terms of severity of ECF clinical disease, an index lower than 0.99 is considered to be a non-reactor to challenge, 0.99-3.99 a mild reaction, 3.99-5.99 a moderate reaction and 6.0 and above as a severe reaction. Animals with an index of 5.99 and lower are classified as immune to ECF. Thus, 5 cattle in group 7 and 12 in group 6 were classified as immune to ECF (33% and 80%, respectively). In group 6, six of the 12 ECF immune cattle had an ECF clinical index of less than two, whereas in group 7 only one of the six immune cattle had an index of less than three. Cattle were treated with buparvaquone when animals reached an ECF index of 6.5, the humane endpoint. There is a statistically significant difference in immunity to ECF between group 6 and 7 cattle (p = 0.025) and in ECF score indices (p-value = 0.003). In this experiment, the sporozoite challenge was calculated to be ~LD_70_.

**Table 5:** Summary of protection results and statistical analysis of protection and ECF score differences between groups 6 and 7.

Group 6	ECF score	S/I	Group 7	ECF score	S/I
BP084	3.68	I	BP102	3.82	I
BP085	5.71	I	BP103	2.97	I
BP086	6.5*	S	BP104	6.5*	S
BP087	0.60	I	BP105	5.73	I
BP088	6.5*	S	BP106	6.5*	S
BP089	1.08	I	BP108	6.5*	S
BP090	3.78	I	BP109	6.5*	S
BP091	1.75	I	BP110	6.5*	S
BP092	5.60	I	BP111	6.5*	S
BP094	1.32	I	BP112	5.46	I
BP095	4.81	I	BP113	6.5*	S
BP097	6.5*	S	BP096	6.5*	S
BP098	2.90	I	BP115	4.88	I
BP099	0.47	I	BP116	6.04	S
BP119	0.60	I	BP118	6.5*	S
Median	3.680		Median	6.5	
Analysis of protection (yes-1/no-0)					
Fisher’s exact test		p-value = 0.025			
		**Group 6**	**Group 7**		
	Immunity level	12/15 (80%)	5/15 (33.3%)		
	Exact 95% CI	51.9%, 95.7%	11.8%, 61.6%		
Analysis of ECF scores					
Permutation test of independence		p-value = 0.003			

*Final ECF score set to 6.5 (humane-end point).

S., susceptible; I., immune, CI. Confidence interval.

## 4 Discussion

Computationally designed nanoparticles have recently emerged as a promising platform for multivalent display of one or more antigens. In most cases reported to date, these scaffolds have been used to display viral glycoprotein antigens, including RSV F (21), HIV-1 Env (11, 14, 15, 42), SARS-CoV-2 Spike (18) and its receptor binding domain (23), EBV gH/gL (43), and influenza hemagglutinin (19). Here we evaluated whether three different computationally designed nanoparticles could also be effective in enhancing the immunogenicity of a polypeptide candidate vaccine antigen from a protozoan parasite that affects cattle. We found that one nanoparticle immunogen, p67C-I53-50, was superior to the soluble antigen s-p67C and to the other two nanoparticles tested, p67C-I32-28 and p67C-I32-19, in priming p67C-specific and sporozoite neutralizing antibody responses, as well as IFN-γ CD4^+^ T-cell responses.

The enhanced potency of p67C when displayed on I53-50 is similar to previous studies comparing I53-50-based nanoparticle immunogens to soluble antigens (14, 21, 23). p67C-I53-50 also induced stronger immune responses than HBcAg-p67**C** VLPs. Our study provides new information on the immunogenicity of the I32-28 scaffold. Although not as potent as the I53-50, both promote antibody switching of the bovine antibody response judged by the presence of IgG2 antibodies, whereas only IgG1 antibodies were induced by the I32-19 scaffold. p67C is a poorly immunogenic protein and it has been useful as a model antigen to evaluate different nanoparticle-based antigen delivery vehicles. Although preliminary, our data suggest that the I53-50 nanoparticle scaffold could be useful in improving the potency of livestock vaccines, especially as it can be easily used to co-display multiple antigens (23). It is also a highly thermostable molecule (26) and can be lyophilized, making it a good platform for use in remote rural areas in low- and middle-income-countries where the delivery of vaccines is a challenge due to inadequate cold chains.

The differences we observed in the immunogenicity of the three p67C nanoparticle immunogens we tested are intriguing. A unique feature of computationally designed nanoparticle scaffolds is that they allow specific structural features to be precisely and accurately varied, but there have been few studies to date that directly compare the same antigen displayed on different nanoparticle scaffolds. Where these have been done, higher valency, i.e., the number of copies of the antigen displayed on the nanoparticle immunogen, appears to correlate with higher immunogenicity (43, 44). Furthermore, I53-50 nanoparticles displaying prefusion human RSV F-protein on 33%, 67%, and 100% of the 20 nanoparticle trimers elicited antibody responses that increased in magnitude with increasing valency (21). Nevertheless, higher valency does not always lead to higher immunogenicity, as comparing the results obtained here to a previous study in which 240 copies of p67C were displayed on HBcAg VLPs suggests that p67C-I53-50 is a more potent scaffold for display of this antigen.

In the present study, each of the three computationally designed nanoparticles we directly compared displayed 60 copies of p67C, so the difference in their immunological performance must be attributed to other factors. Although somewhat speculative, it is possible that I53-50 is more physically stable *in vivo* than the other two nanoparticles, and this may account for its higher immunogenicity. We note that I32-19 and I32-28 both showed signs of instability, particularly when assembled *in vitro*. Furthermore, I53-50 has been previously noted to be an unusually stable assembly, a property that has been shown in some cases to improve the stability of antigens genetically fused to its components (21, 26). Additional studies will be required to more broadly define the role of nanoparticle scaffold stability in the immunogenicity of nanoparticle vaccines. A recent study along these lines that investigated antigen stability *in vivo* highlighted the role of proteases in defining immunogenicity and the epitope specificity of vaccine-elicited antibodies (45).

Mapping of epitopes on p67 has been restricted to Pepscan analyses of the reactivity of bovine antibody responses and p67-specific murine mAbs to synthetic 15-mer overlapping peptides (40). Unfortunately, little is known about conformational epitopes on p67. Immunization of cattle with full-length p67 resulted in a dominant recognition of peptides 79, 80, and 81 encoded within p67C, whereas peptides 78, 79, and 80 are recognized by sporozoite neutralizing mAbs. In this study, immunization of p67C as a soluble antigen or in a nanoparticle format also resulted in a poor response to peptide 78, a maintained antibody reactivity with peptide 79 and 80, and a novel reactivity with peptide 74 and 75. Peptide 75 is recognized by a non-neutralizing mAb. The functional consequences of these peptide reactivity patterns will require further study.

As p67C-I53-50 nanoparticles were easier to work with and induced superior immune responses to p67C-I32-28 and p67C-I32-19, we only carried out an experimental vaccine trial with the former, using three immunizations with 140 μg of antigen per dose. Relative to control cattle, the p67C-I53-50 nanoparticle exhibited a vaccine efficacy close to 50%. This may appear low but represents promising data as the median ECF index in the control group was 6.50, while that in the experimental group was 3.68 (p-value 0.03). The efficacy data is like that seen in experimental vaccine trials with s-p67C with an antigen dose of 450 μg (33) and the efficacy induced by a combination of HBcAg-p67C and SV-p67C (35). As ECF in the control cohorts in each of the experiments varied from an LD70~LD90 it is difficult to make direct comparisons between these small-scale experiments. Nevertheless, the data suggests that I53-50 permits dose sparing of the p67C antigen and it can replace the combination of two nanoparticles, HBcAg-p67C and SV-p67C, used previously.

Although p67C remains a promising candidate vaccine antigen, the combinatorial effect of adding additional targets of sporozoite neutralizing antibodies to an experimental vaccine awaits testing. For example, we know that the p67 antigen contains additional sporozoite neutralizing epitopes (32, 40), and we have identified other sporozoite antigens which induce neutralizing antibodies (46). The higher immunogenicity associated with the I53-50 scaffold and the ease of production and manipulation of the nanoparticles strongly support the use of this platform in further studies and could lead to the development of more effective vaccines for ECF. Conversely, application of such computationally designed nanoparticles to other livestock diseases for which protective antigens are well-defined (47–49), or can be identified *via* homology to human pathogens (50–52), may be a useful strategy for efficiently applying this novel vaccine delivery technology to improve global health and well-being.

## Data availability statement

The original contributions presented in the study are included in the article/supplementary material. Further inquiries can be directed to the corresponding authors.

## Ethics statement

The animal study was reviewed and approved by International Livestock Research Institute (ILRI) - Institutional Animal Care and Use Committee (IACUC).

## Author contributions

AL and VN designed and supervised all *in vivo* experiments and *ex vivo* analysis. AL, BF, VN and NK performed the final data analysis and manuscript writing. NK designed the p67C-nanoparticles. HK, EK, GT, AS, BF expressed, purified, characterized and quality controlled the nanoparticle formation. RG, NC, RO, CM and SM performed the *ex vivo* analysis of animal samples, under AL supervision. EP and NN performed the statistical analysis. All authors contributed to the article and approved the submitted version.

## References

[1] Kanekiyo M Ellis D King NP. New vaccine design and delivery technologies. J Infect Dis (2019) 219(Suppl_1):S88–96. doi: 10.1093/infdis/jiy745 PMC6452296 30715361

[2] Raeven RHM van Riet E Meiring HD Metz B Kersten GFA. Systems vaccinology and big data in the vaccine development chain. Immunol [Internet] (2019) 156(1):33–46. doi: 10.1111/imm.13012 PMC6283655 30317555

[3] Pulendran B Li S Nakaya HI. Systems vaccinology. Immunity (2010) 33(4):516–29. doi: 10.1016/j.immuni.2010.10.006 PMC3001343 21029962

[4] Dungu B Lubisi BA Ikegami T. Rift valley fever vaccines: Current and future needs. Curr Opin Virol (2018) 29:8–15. doi: 10.1016/j.coviro.2018.02.001 29514112

[5] Choi B Kim H Choi H Kang S. Protein cage nanoparticles as delivery nanoplatforms. Adv Exp Med Biol (2018) 1064:27–43. doi: 10.1007/978-981-13-0445-3_2 30471024

[6] King NP Sheffler W Sawaya MR Vollmar BS Sumida JP André I. Computational design of self-assembling protein nanomaterials with atomic level accuracy. Science (80-) (2012) 336:1171–4. doi: 10.1126/science.1219364 PMC4138882 22654060

[7] King NP Bale JB Sheffler W McNamara DE Gonen S Gonen T. Accurate design of co-assembling multi-component protein nanomaterials. Nature (2014) 510(7503):103–8. doi: 10.1038/nature13404 PMC4137318 24870237

[8] Divine R Dang HV Ueda G Fallas JA Vulovic I Sheffler W. Designed proteins assemble antibodies into modular nanocages. Science (2021) 372(6537). doi: 10.1126/science.abd9994 PMC8592034 33795432

[9] Bale JB Gonen S Liu Y Sheffler W Ellis D Thomas C. Accurate design of megadalton-scale two-component icosahedral protein complexes. Science (80-) (2016) 353(6297):389–94. doi: 10.1126/science.aaf8818 PMC5485857 27463675

[10] Hsia Y Bale JB Gonen S Shi D Sheffler W Fong KK. Design of a hyperstable 60-subunit protein dodecahedron. Nature (2016) 535(7610):136–9. doi: 10.1038/nature18010 PMC4945409 27309817

[11] Ueda G Antanasijevic A Fallas JA Sheffler W Copps J Ellis D. Tailored design of protein nanoparticle scaffolds for multivalent presentation of viral glycoprotein antigens. Elife (2020) 9:1–30. doi: 10.7554/eLife.57659 PMC7402677 32748788

[12] Zakeri B Fierer JO Celik E Chittock EC Schwarz-Linek U Moy VT. Peptide tag forming a rapid covalent bond to a protein, through engineering a bacterial adhesin. Proc Natl Acad Sci U.S.A. (2012) 109(12):E690–7. doi: 10.1073/pnas.1115485109 PMC3311370 22366317

[13] Veggiani G Zakeri B Howarth M. Superglue from bacteria: Unbreakable bridges for protein nanotechnology. Trends Biotechnol (2014) 32(10):506–12. doi: 10.1016/j.tibtech.2014.08.001 PMC4281928 25168413

[14] Brouwer PJM Antanasijevic A Berndsen Z Yasmeen A Fiala B Bijl TPL. Enhancing and shaping the immunogenicity of native-like HIV-1 envelope trimers with a two-component protein nanoparticle. Nat Commun (2019) 10(1). doi: 10.1038/s41467-019-12080-1 PMC6753213 31537780

[15] Brouwer PJM Antanasijevic A de Gast M Allen JD Bijl TPL Yasmeen A. Immunofocusing and enhancing autologous tier-2 HIV-1 neutralization by displaying env trimers on two-component protein nanoparticles. NPJ Vaccines (2021) 6(1). doi: 10.1038/s41541-021-00285-9 PMC7873233 33563983

[16] Perotti M Marcandalli J Demurtas D Sallusto F Perez L. Rationally designed human cytomegalovirus gB nanoparticle vaccine with improved immunogenicity. PloS Pathog (2020) 16(12). doi: 10.1371/journal.ppat.1009169 PMC7794029 33370407

[17] Cohen AA Gnanapragasam PNP Lee YE Hoffman PR Ou S Kakutani LM. Mosaic nanoparticles elicit cross-reactive immune responses to zoonotic coronaviruses in mice. Science (2021) 371(6530):735–41. doi: 10.1126/science.abf6840 PMC7928838 33436524

[18] Brouwer PJM Brinkkemper M Maisonnasse P Dereuddre-Bosquet N Grobben M Claireaux M. Two-component spike nanoparticle vaccine protects macaques from SARS-CoV-2 infection. Cell (2021) 184(5):1188–1200.e19. doi: 10.1016/j.cell.2021.01.035 33577765 PMC7834972

[19] Boyoglu-Barnum S Ellis D Gillespie RA Hutchinson GB Park YJ Moin SM. Quadrivalent influenza nanoparticle vaccines induce broad protection. Nature (2021) 592(7855):623–8. doi: 10.1038/s41586-021-03365-x PMC8269962 33762730

[20] Ellis D Brunette N Crawford KHD Walls AC Pham MN Chen C. Stabilization of the SARS-CoV-2 spike receptor-binding domain using deep mutational scanning and structure-based design. Front Immunol (2021) 12. doi: 10.3389/fimmu.2021.710263 PMC8276696 34267764

[21] Marcandalli J Fiala B Ols S Perotti M de van der Schueren W Snijder J. Induction of potent neutralizing antibody responses by a designed protein nanoparticle vaccine for respiratory syncytial virus. Cell (2019) 176(6):1420–1431.e17. doi: 10.1016/j.cell.2019.01.046 30849373 PMC6424820

[22] Dalvie NC Rodriguez-Aponte SA Hartwell BL Tostanoski LH Biedermann AM Crowell LE. Engineered SARS-CoV-2 receptor binding domain improves manufacturability in yeast and immunogenicity in mice. Proc Natl Acad Sci U.S.A. (2021) 118(38). doi: 10.1073/pnas.2106845118 PMC8463846 34493582

[23] Walls AC Fiala B Schäfer A Wrenn S Pham MN Murphy M. Elicitation of potent neutralizing antibody responses by designed protein nanoparticle vaccines for SARS-CoV-2. Cell (2020) 183(5):1367–1382.e17. doi: 10.1016/j.cell.2020.10.043 33160446 PMC7604136

[24] Bruun TUJ Andersson AMC Draper SJ Howarth M. Engineering a rugged nanoscaffold to enhance plug-and-Display vaccination. ACS Nano (2018) 12(9):8855–66. doi: 10.1021/acsnano.8b02805 PMC6158681 30028591

[25] Kang YF Sun C Zhuang Z Yuan RY Zheng Q Li JP. Rapid development of SARS-CoV-2 spike protein receptor-binding domain self-assembled nanoparticle vaccine candidates. ACS Nano (2021) 15(2):2738–52. doi: 10.1021/acsnano.0c08379 33464829

[26] Wargacki AJ Wörner TP van de Waterbeemd M Ellis D Heck AJR King NP. Complete and cooperative in vitro assembly of computationally designed self-assembling protein nanomaterials. Nat Commun (2021) 12(1). doi: 10.1038/s41467-021-21251-y PMC7873210 33563988

[27] Norval R Perry B Young A. The epidemiology of theileriosis in africa. the epidemiology of theileriosis in Africa. London: Academic Press Limited (1992).

[28] Perry BD. The control of East coast fever of cattle by live parasite vaccination: A science-to-impact narrative. One Heal (2016) 2:103–14. doi: 10.1016/j.onehlt.2016.07.002 PMC5441314 28616483

[29] Muraguri GR McLeod A McDermott JJ Taylor N. The incidence of calf morbidity and mortality due to vector-borne infections in smallholder dairy farms in kwale district, Kenya. Vet Parasitol (2005) 130(3–4):305–15. doi: 10.1016/j.vetpar.2004.11.026 15885914

[30] Di Giulio G Lynen G Morzaria S Oura C Bishop R. Live immunization against East coast fever–current status. Trends Parasitol (2009) 25(2):85–92. doi: 10.1016/j.pt.2008.11.007 19135416

[31] Nene V Morrison WI. Approaches to vaccination against theileria parva and theileria annulata. Parasite Immunol (2016) 38(12):724–34. doi: 10.1111/pim.12388 PMC5299472 27647496

[32] Bishop R Nene V Staeyert J Rowlands J Nyanjui J Osaso J. Immunity to East coast fever in cattle induced by a polypeptide fragment of the major surface coat protein of theileria parva sporozoites. Vaccine 21(11–12):1205–12. doi: 10.1016/S0264-410X(02)00621-7 12559799

[33] Lacasta A Mwalimu S Kibwana E Saya R Awino E Njoroge T. Immune parameters to p67C antigen adjuvanted with ISA206VG correlate with protection against East coast fever. Vaccine (2018) 36:1389–7. doi: 10.1016/j.vaccine.2018.01.087 PMC5835154 29429808

[34] Musoke A Rowlands J Nene V Nyanjui J Katende J Spooner P. Subunit vaccine based on the p67 major surface protein of theileria parva sporozoites reduces severity of infection derived from field tick challenge. Vaccine (2005) 23(23):3084–95. doi: 10.1016/j.vaccine.2004.09.039 15811656

[35] Lacasta A Mody KT De Goeyse I Yu C Zhang J Nyagwange J. Synergistic effect of two nanotechnologies enhances the protective capacity of the theileria parva sporozoite p67C antigen in cattle. J Immunol (2021) 206:686–99. doi: 10.4049/jimmunol.2000442 PMC7851744 33419770

[36] Bachmann MF Jennings GT. Vaccine delivery: A matter of size, geometry, kinetics and molecular patterns. Nat Rev Immunol (2010) 10(11):787–96. doi: 10.1038/nri2868 20948547

[37] Katende J Morzaria S Toye P Skilton R Nene V Nkonge C. An enzyme-linked immunosorbent assay for detection of theileria parva antibodies in cattle using a recombinant polymorphic immunodominant molecule. Parasitol Res (1998) 84(5):408–16. doi: 10.1007/s004360050419 9610640

[38] Rowlands GJ Musoke AJ Morzaria SP Nagda SM Ballingall KT McKeever DJ. A statistically derived index for classifying East coast fever reactions in cattle challenged with theileria parva under experimental conditions. Parasitology (2000) 120(4):371–81. doi: 10.1017/s0031182099005600 10811278

[39] Hjerrild KA Jin J Wright KE Brown RE Marshall JM Labbé GM. Production of full-length soluble plasmodium falciparum RH5 protein vaccine using a drosophila melanogaster Schneider 2 stable cell line system. Sci Rep (2016) 6. doi: 10.1038/srep30357 PMC4960544 27457156

[40] Nene V Gobright E Bishop R Musoke A. Linear peptide specificity of bovine antibody responses to p67 of *Theileria parva* and sequence diversity of Sporozoite-neutralizing Epitopes: Implications for a vaccine. (1999) 67(3):1261–6. doi: 10.1128/IAI.67.3.1261-1266.1999 PMC96455 10024569

[41] R Core Team. R: A language and environment for statistical computing. Vienna, Austria: R Foundation for Statistical Computing (2022).

[42] Antanasijevic A Ueda G Brouwer PJM Copps J Huang D Allen JD. Structural and functional evaluation of *de novo*-designed, two-component nanoparticle carriers for HIV env trimer immunogens. PloS Pathog (2020) 16(8). doi: 10.1371/journal.ppat.1008665 PMC7418955 32780770

[43] Malhi H Homad LJ Wan YH Poudel B Fiala B Borst AJ. Immunization with a self-assembling nanoparticle vaccine displaying EBV gH/gL protects humanized mice against lethal viral challenge. Cell Rep Med (2022) 3(6):100658. doi: 10.1016/j.xcrm.2022.100658 35705092 PMC9245003

[44] Kato Y Abbott RK Freeman BL Haupt S Groschel B Silva M. Multifaceted effects of antigen valency on b cell response composition and differentiation *In vivo* . Immunity (2020) 53(3):548–563.e8. doi: 10.1016/j.immuni.2020.08.001 32857950 PMC7451196

[45] Aung A Cui A Soleimany AP Bukenya M Lee H Cottrell CA. Spatially regulated protease activity in lymph nodes renders b cell follicles a sanctuary for retention of intact antigens. bioRxiv (2021). doi: 10.1101/2021.11.15.468669

[46] Nyagwange J Nene V Mwalimu S Henson S Steinaa L Nzau B. Antibodies to in silico selected GPI-anchored theileria parva proteins neutralize sporozoite infection *in vitro* . Vet Immunol Immunopathol (2018) 199:8–14. doi: 10.1016/j.vetimm.2018.03.004 29678234 PMC5956992

[47] Zhou Y Jiang S Du L. Prospects for a MERS-CoV spike vaccine. Expert Rev Vaccines (2018) 17(8):677–86. doi: 10.1080/14760584.2018.1506702 PMC6355461 30058403

[48] Kumar N Barua S Riyesh T Tripathi BN. Advances in peste des petits ruminants vaccines. Vet Microbiol (2017) 206:91–101. doi: 10.1016/j.vetmic.2017.01.010 28161212 PMC7130925

[49] Riffault S Hägglund S Guzman E Näslund K Jouneau L Dubuquoy C. A single shot pre-fusion-stabilized bovine rsv f vaccine is safe and effective in newborn calves with maternally derived antibodies. Vaccines (2020) 8:231. doi: 10.3390/vaccines8020231 32443437 PMC7349975

[50] Chung JY Thone MN Kwon YJ. COVID-19 vaccines: The status and perspectives in delivery points of view. Adv Drug Delivery Rev (2021) 170:1–25. doi: 10.1016/j.addr.2020.12.011 PMC7759095 33359141

[51] Du L He Y Zhou Y Liu S Zheng BJ Jiang S. The spike protein of SARS-CoV–a target for vaccine and therapeutic development. Nat Rev Microbiol (2009) 7(3):226–36. doi: 10.1038/nrmicro2090 PMC2750777 19198616

[52] Mejias A Rodríguez-Fernández R Oliva S Peeples ME Ramilo O. The journey to a respiratory syncytial virus vaccine. Ann Allergy Asthma Immunol (2020) 125(1):36–46. doi: 10.1016/j.anai.2020.03.017 32217187 PMC7311299

