# Supplementary material for: Design and immunological evaluation of two-component protein nanoparticle vaccines for East Coast fever
Source: Front Immunol. 2023 Jan 13;13:1015840. doi: 10.3389/fimmu.2022.1015840 (PMC9880323; doi:10.3389/fimmu.2022.1015840)
Supplement: Supplementary file 1 [file DataSheet_1.docx]

Supplementary Material

**Table S1 | Amino acid sequence of all novel proteins used in this study.**

>p67C-I53-50 (co-expressed)

MGTGGGSLRGLDLSEEEVKKILDEIVKDPSDGELGLGDLSDPSGRSSERQPSLGPSLVITDGQAGPTIVSPTGPTIAAGGEHGGSGGSGGKMEELFKKHKIVAVLRANSVEEAIEKAVAVFAGGVHLIEITFTVPDADTVIKALSVLKEKGAIIGAGTVTSVEQCRKAVESGAEFIVSPHLDEEISQFCKEKGVFYMPGVMTPTELVKAMKLGHDILKLFPGEVVGPQFVKAMKGPFPNVKFVPTGGVNLDNVCEWFKAGVLAVGVGDALVKGDPDEVREKAKKFVEKIRGCTE

MNQHSHKDHETVRIAVVRARWHAEIVDACVSAFEAAMRDIGGDRFAVDVFDVPGAYEIPLHARTLAETGRYGAVLGTAFVVDGGIYDHEFVASAVIDGMMNVQLDTGVPVLSAVLTPHEYEDSDADTLLFLALFAVKGMEAARACVEILAAREKIAAGSLEHHHHHH

>p67C-I32-19 (co-expressed)

MGTGGGSLRGLDLSEEEVKKILDEIVKDPSDGELGLGDLSDPSGRSSERQPSLGPSLVITDGQAGPTIVSPTGPTIAAGGEHGGSDLQKLQRFSTCDISDGLLNVYNIPTGGYFPNLTAISPPQNSSIVGTAYTVLFAPIDDPRPAVNYIDSVPPNSILVLALEPHLQSQFHPFIKITQAMYGGLMSTRAQYLKSNGTVVFGRIRDVDEHRTLNHPVFAYGVGSCAPKAVVKAVGTNVQLKILTSDGVTQTICPGDYIAGDNNGIVRIPVQETDISKLVTYIEKSIEVDRLVSEAIKNGLPAKAAQTARRMVLKDYI

MSGMRVYLGADHAGYELKQAIIAFLKMTGHEPIDCGALRYDADDDYPAFCIAAATRTVADPGSLGIVLGGSGNGEQIAANKVPGARCALAWSVQTAALAREHNNAQLIGIGGRMHTLEEALRIVKAFVTTPWSKAQRHQRRIDILAEYERTHEAPPVPGAPALEHHHHHH

>p67C-I32-28 (co-expressed)

MGTGGGSLRGLDLSEEEVKKILDEIVKDPSDGELGLGDLSDPSGRSSERQPSLGPSLVITDGQAGPTIVSPTGPTIAAGGEHGGDDARIAAIGDVDELNSQIGVLLAEPLPDDVRAALSAIQHDLFDLGGELCIPGHAAITEDHLLRLALWLVHYNGQLPPLEEFILPGGARGAALAHVCRTVCRRAERSIKALGASEPLNIAPAAYVNLLSDLLFVLARVLNRAAGGADVLWDRTRAH

MILSAEQSFTLRHPHGQAAALAFVREPAAALAGVQRLRGLDSDGEQVWGELLVRVPLLGEVDLPFRSEIVRTPQGAELRPLTLTGERAWVAVSGQATAAEGGEMAFAFQFQAHLATPEAEGEGGAAFEVMVQAAAGVTLLLVAMALPQGLAAGLPPALEHHHHHH

>p67C-I53-50A

MGTGGGSLRGLDLSEEEVKKILDEIVKDPSDGELGLGDLSDPSGRSSERQPSLGPSLVITDGQAGPTIVSPTGPTIAAGGEHGGSGGSGGKIEELFKKHKIVAVLRANSVEEAIEKAVAVFAGGVHLIEITFTVPDADTVIKALSVLKEKGAIIGAGTVTSVEQCRKAVESGAEFIVSPHLDEEISQFCKEKGVFYMPGVMTPTELVKAMKLGHDILKLFPGEVVGPQFVKAMKGPFPNVKFVPTGGVNLDNVCEWFKAGVLAVGVGDALVKGDPDEVREKAKKFVEKIRGCTELEHHHHHH

>p67C-I32-19A

MGTGGGSLRGLDLSEEEVKKILDEIVKDPSDGELGLGDLSDPSGRSSERQPSLGPSLVITDGQAGPTIVSPTGPTIAAGGEHGGSDLQKLQRFSTCDISDGLLNVYNIPTGGYFPNLTAISPPQNSSIVGTAYTVLFAPIDDPRPAVNYIDSVPPNSILVLALEPHLQSQFHPFIKITQAMYGGLMSTRAQYLKSNGTVVFGRIRDVDEHRTLNHPVFAYGVGSCAPKAVVKAVGTNVQLKILTSDGVTQTICPGDYIAGDNNGIVRIPVQETDISKLVTYIEKSIEVDRLVSEAIKNGLPAKAAQTARRMVLKDYILEHHHHH

>p67C-I32-28A

MGTGGGSLRGLDLSEEEVKKILDEIVKDPSDGELGLGDLSDPSGRSSERQPSLGPSLVITDGQAGPTIVSPTGPTIAAGGEHGGDDARIAAIGDVDELNSQIGVLLAEPLPDDVRAALSAIQHDLFDLGGELCIPGHAAITEDHLLRLALWLVHYNGQLPPLEEFILPGGARGAALAHVCRTVCRRAERSIKALGASEPLNIAPAAYVNLLSDLLFVLARVLNRAAGGADVLWDRTRAHLEHHHHHH

>I53-50B.4PT1

MNQHSHKDHETVRIAVVRARWHAEIVDACVSAFEAAMRDIGGDRFAVDVFDVPGAYEIPLHARTLAETGRYGAVLGTAFVVNGGIYRHEFVASAVINGMMNVQLNTGVPVLSAVLTPHNYDKSKAHTLLFLALFAVKGMEAARACVEILAAREKIAA

>I32-19B

MSGMRVYLGADHAGYELKQAIIAFLKMTGHEPIDCGALRYDADDDYPAFCIAAATRTVADPGSLGIVLGGSGNGEQIAANKVPGARCALAWSVQTAALAREHNNAQLIGIGGRMHTLEEALRIVKAFVTTPWSKAQRHQRRIDILAEYERTHEAPPVPGAPALEHHHHHH

>I32-28B

MILSAEQSFTLRHPHGQAAALAFVREPAAALAGVQRLRGLDSDGEQVWGELLVRVPLLGEVDLPFRSEIVRTPQGAELRPLTLTGERAWVAVSGQATAAEGGEMAFAFQFQAHLATPEAEGEGGAAFEVMVQAAAGVTLLLVAMALPQGLAAGLPPALEHHHHHH

**Table S2 | to 15-mer biotinylated peptides overlapping in 7 amino acids used in peptide specificity ELISA.**

| Peptide name* | Peptide sequence | Peptide length |
| --- | --- | --- |
| Pin 73 | RAAGTGGGSLRGLDL | 15 |
| Pin 74 | SLRGLDLSEEEVKKI | 15 |
| Pin 75 | EEEVKKILDEIVKDP | 15 |
| Pin 76 | DEIVKDPSDGELGLG | 15 |
| Pin 77 | DGELGLGDLSDPSGR | 15 |
| Pin 78 | LSDPSGRSSERQPSL | 15 |
| Pin 79 | SERQPSLGPSLVITD | 15 |
| Pin 80 | PSLVITDGQAGPTIV | 15 |
| Pin 81 | QAGPTIVSPTGPTIA | 15 |
| Pin 82 | PTGPTIAAGGE | 11 |

*Peptide name matching previous publication by Nene et al., 1999 (DOI: [10.1128/IAI.67.3.1261-1266.1999](https://doi.org/10.1128/iai.67.3.1261-1266.1999))

**Table S3 | Summary of p67C-specific antibody titers (μg/ml) in animals from group 1 to 5 (immunogenicity studies).**

| **Group** | **Animal ID** | **Day 14** | **Day 28** | **Day 42** | **Day 56** | **Day 70** | **Day 77** | **Day 91** | **Day 105** | **Day 119** | **Day 133** |
| --- | --- | --- | --- | --- | --- | --- | --- | --- | --- | --- | --- |
| Group 1 | BN035 | 463.58 | 240.86 | 347.53 | 990.60 | 896.14 | 607.02 | 569.75 | 330.78 | 162.79 | 60.38 |
| (p67C-I32-19) | BN050 | 240.25 | 113.73 | 94.36 | 486.67 | 541.81 | 412.43 | 460.86 | 30.07 | 125.81 | 148.45 |
|  | BN064 | 116.56 | 20.23 | 106.40 | 625.50 | 574.67 | 251.59 | 116.85 | 98.77 | 25.34 | 27.95 |
| Group 2 | BN049 | 356.22 | 224.12 | 5042.68 | 5184.43 | 3052.92 | 1342.16 | 912.17 | 1081.70 | 560.26 | 593.07 |
| (p67C-I32-28) | BN055 | 0.00 | 287.30 | 2447.37 | 2657.72 | 1985.67 | 1255.10 | 523.50 | 800.02 | 367.73 | 198.43 |
|  | BN068 | 0.00 | 40.57 | 607.05 | 1636.23 | 2610.59 | 1596.97 | 800.86 | 1211.07 | 438.53 | 236.57 |
| Group 3 | BN043 | 46.92 | 100.53 | 4192.14 | 1879.41 | 3651.00 | 2292.79 | 938.95 | 737.70 | 142.09 | 191.96 |
| (p67C-I53-50) | BN054 | 131.52 | 130.92 | 4864.02 | 3517.18 | 6743.43 | 5431.88 | 1877.37 | 2461.82 | 1034.45 | 317.51 |
|  | BN058 | 341.19 | 492.19 | 6154.55 | 5931.73 | 166639.73 | 18508.03 | 7278.45 | 3693.52 | 5256.64 | 2319.06 |
| Group 4 | BM005 |  | 54.71 | 268.38 | 225.83 | 918.28 | 556.31 | 670.40 | 399.02 | 361.04 | 210.42 |
| (s-p67C) | BM062 |  | 35.79 | 194.75 | 126.87 | 617.73 | 347.94 | 353.64 | 197.80 | 217.31 | 184.29 |
|  | BM065 |  | 33.32 | 72.69 | 64.48 | 410.36 | 343.14 | 274.43 | 203.45 | 154.32 | 145.52 |
| Group 5 | BM135 | 308.56 | 671.29 | 1591.58 | 1123.78 | 1419.99 | 4569.33 | 1784.48 | 974.08 | 780.11 | 653.94 |
| (HBcAg-p67C) | BM162 | 305.89 | 670.40 | 1703.98 | 1263.66 | 1717.18 | 2209.45 | 965.28 | 568.33 | 488.53 | 388.48 |
|  | BM203 | 1012.63 | 703.12 | 1863.35 | 1503.15 | 2457.80 | 4018.22 | 1831.86 | 1240.93 | 1041.57 | 790.38 |

Grey boxes: non-available data.

**Table S4 | Summary of p67C-specific antibody titers (μg/ml) in animals from group 6 (challenge experiment).**

| **Group** | **Animal ID** | **Day 14** | **Day 28** | **Day 42** | **Day 56** | **Day 70** | **Day 77** | **Day 84** | **Day 91** |
| --- | --- | --- | --- | --- | --- | --- | --- | --- | --- |
| Group 6 | BP084 | 60.17 | 243.10 | 2287.37 | 1022.02 | 1846.22 | 6149.55 | 844.10 | 838.43 |
| (p67C-I53-50) | BP085 | 119.20 | 158.50 | 5850.90 | 2229.92 | 3525.85 | 16088.27 | 1058.69 | 1230.86 |
|  | BP086 | 55.76 | 140.44 | 1801.62 | 925.76 | 1451.56 | 672.16 | 545.60 | 569.50 |
|  | BP087 | 101.42 | 180.67 | 1362.82 | 980.89 | 3694.88 | 16720.74 | 941.88 | 1069.46 |
|  | BP088 | 100.21 | 177.96 | 2470.79 | 1330.60 | 3238.24 | 12111.66 | 867.27 | 941.21 |
|  | BP089 | 34.64 | 82.24 | 1787.92 | 781.12 | 909.23 | 5823.08 | 441.32 | 508.13 |
|  | BP090 | 60.80 | 167.21 | 1321.42 | 521.81 | 3174.82 | 11052.98 | 685.03 | 765.21 |
|  | BP091 | 42.90 | 80.43 | 783.57 | 401.31 | 883.03 | 7380.33 | 622.94 | 459.51 |
|  | BP092 | 46.73 | 177.03 | 1863.45 | 772.27 | 1281.36 | 7861.16 | 684.27 | 542.56 |
|  | BP094 | 110.20 | 233.60 | 1987.68 | 674.93 | 1196.63 | 8154.07 | 406.45 | 450.26 |
|  | BP095 | 12.04 | 71.74 | 760.72 | 461.44 | 1470.60 | 7380.33 | 796.69 | 622.09 |
|  | BP097 | 225.58 | 215.32 | 3211.66 | 1719.98 | 1896.84 | 794.71 | 1277.54 | 1079.55 |
|  | BP098 | 113.81 | 187.64 | 1540.35 | 1059.39 | 1995.69 | 957.49 | 660.18 | 382.05 |
|  | BP099 | 152.50 | 229.42 | 1870.84 | 1602.53 | 4365.36 | 2639.00 | 1465.49 | 1539.32 |
|  | BP119 | 96.79 | 191.06 | 2765.93 | 1676.60 | 3344.16 | 2172.89 | 1804.90 | 1414.93 |
